# Home‐ and community‐level predictors of social connection in nursing home residents: A scoping review

**DOI:** 10.1002/hsr2.743

**Published:** 2022-07-20

**Authors:** Sara Clemens, Katelynn Aelick, Jessica Babineau, Monica Bretzlaff, Cathleen Edwards, Josie‐Lee Gibson, Debbie Hewitt Colborne, Andrea Iaboni, Dee Lender, Denise Schon, Ellen Snowball, Katherine S. McGilton, Jennifer Bethell

**Affiliations:** ^1^ KITE Research Institute Toronto Rehabilitation Institute‐University Health Network Toronto Ontario Canada; ^2^ Behavioural Supports Ontario Provincial Coordinating Office North Bay Regional Health Centre North Bay Ontario Canada; ^3^ Library and Information Services University Health Network Toronto Ontario Canada; ^4^ The Institute for Education Research University Health Network Toronto Ontario Canada; ^5^ Family Councils Ontario Toronto Ontario Canada; ^6^ Ontario Association of Residents' Councils Newmarket Ontario Canada; ^7^ Department of Psychiatry University of Toronto Toronto Ontario Canada; ^8^ Lakeside Long‐Term Care Centre Family Council Toronto Ontario Canada; ^9^ Lawrence S. Bloomberg Faculty of Nursing University of Toronto Toronto Ontario Canada; ^10^ Institute of Health Policy, Management and Evaluation University of Toronto Toronto Ontario Canada

**Keywords:** nursing home design and construction, personnel staffing and scheduling, social behavior, social isolation

## Abstract

**Background and Aims:**

Social connection is associated with better physical and mental health and is an important aspect of the quality of care for nursing home residents. The primary objective of this scoping review was to answer the question: what nursing home and community characteristics have been tested as predictors of social connection in nursing home residents? The secondary objective was to describe the measures of social connection used in these studies.

**Methods:**

We searched MEDLINE(R) ALL (Ovid), CINAHL (EBSCO), APA PsycINFO (Ovid), Scopus, Sociological Abstracts (ProQuest), Embase and Embase Classic (Ovid), Emcare Nursing (Ovid), and AgeLine (EBSCO) for research that quantified associations between nursing home and/or community characteristics and resident social connection. Searches were limited to English‐language articles published from database inception to search date (July 2019) and update (January 2021).

**Results:**

We found 45 studies that examined small‐scale home‐like settings (17 studies), facility characteristics (14 studies), staffing characteristics (11 studies), care philosophy (nine studies), and community characteristics (five studies). Eight studies assessed multiple home or community‐level exposures. The most frequent measures of social connection were study‐specific assessments of social engagement (11 studies), the Index of Social Engagement (eight studies) and Qualidem social relations (six studies), and/or social isolation (five studies) subscales. Ten studies assessed multiple social connection outcomes.

**Conclusion:**

Research has assessed small‐scale home‐like settings, facility characteristics, staffing characteristics, care philosophy, and community characteristics as predictors of social connection in nursing home residents. In these studies, there was no broad consensus on best approach(es) to the measurement of social connection. Further research is needed to build an evidence‐base on how modifiable built environment, staffing and care philosophy characteristics—and the interactions between these factors—impact residents' social connection.

## 1 INTRODUCTION

COVID‐19, and the infection control measures enacted to prevent it, have highlighted the importance of social connection to the health and well‐being of nursing home residents.^1^ Relationships between residents as well as those with family and staff contribute to resident well‐being^2^ and are a key aspect of both quality of life^3^ and quality of care^4^ in nursing homes.

Social connection depends on the existence, roles, and qualities of relationships as well as the sense of connection within these relationships.^5^ Social connection encompasses distinct aspects, including loneliness, social support, and social engagement.^6^ Multiple aspects of social connection have been highlighted for research in nursing homes.^7^

Nursing home design and location have been described as important influences on social connection for residents^8^ and nursing home residents have expressed the importance of designing nursing homes accordingly.^9^ Although nursing home characteristics have been found to impact quality of life,^10^ surprisingly little quantitative research has been published in this area. A 2013 systematic review assessing the impact of nursing home characteristics on overall resident quality of life found 11 studies with mixed results and an inadequate evidence base.^11^ Subsequent reviews focused on quality of life have highlighted the influence of physical environments^12^ and design^13^ for residents with dementia. The impact of community characteristics on resident social connection and quality of life more broadly, is even less clear. The objective of this scoping review is to summarize published research testing nursing home‐ and community‐level predictors of social connection in residents. By identifying gaps in knowledge, this review will inform future research on approaches to building and maintaining social connection among nursing home residents.

## 2 METHODS

Our scoping review was designed to map research evidence on social connection in nursing homes. It followed a published protocol,^14^ used a six‐stage approach^15^, ^16^ and is reported according to the PRISMA Extension for Scoping Reviews.^17^

### 2.1 Step 1: Identifying the research questions

We sought to address the research question: what nursing home and community characteristics have been tested as predictors of social connection in nursing home residents? This question evolved from the needs of knowledge users after completing a scoping review examining the mental health impacts of social connection and potential strategies during COVID‐19.^18^ As a secondary objective, from these studies, we described the measures that were used to assess social connection in nursing home residents.

### 2.2 Step 2: Searching for relevant studies

Published observational and intervention studies were eligible for this review if they met each of these criteria:

- **Population**: reported results from adult residents of nursing homes. Studies conducted in other settings, including assisted living facilities and retirement homes, were not included.
- **Intervention**: delivered at the nursing home or community level or
- **Exposure**: assessed nursing home or community characteristics with an ecological measure (i.e., properties of groups or places).^19^
- **Comparator**: any.
- **Outcome**: reported any quantitative measure of social connection (including social networks, social support, social engagement, social isolation, loneliness, social capital, and social connectedness), including where assessed through quality‐of‐life subscales.

A comprehensive search strategy^14^ was developed with an experienced information specialist. We searched multiple databases in the fields on health sciences and focused on subareas of healthcare such as nursing and allied health. We also explored the social sciences and a multidisciplinary database. The information specialist conducted the search in MEDLINE(R) ALL (in Ovid, including Epub Ahead of Print, In‐Process & Other Non‐Indexed Citations, Ovid MEDLINE[R] Daily) and then translated it into CINAHL (EBSCO), APA PsycINFO (Ovid), Scopus, Sociological Abstracts (Proquest), Embase and Embase Classic (Ovid), Emcare Nursing (Ovid), and AgeLine (EBSCO). See Supporting Information: Appendix A for the Medline search strategy.

Searches were limited to the English language and conducted from the databases' inception through to July 2019 and updated in January 2021. Covidence (www.covidence.org) and EndNote were used to manage the review process, including the deduplication of database results.

### 2.3 Step 3: Selecting studies

As part of the initial review, two reviewers independently screened titles and abstracts then full articles to identify potentially relevant studies (i.e., studies that quantified social connection in nursing home residents). Any disagreements were resolved by a third reviewer. For this subanalysis, two reviewers independently reviewed these full text papers to identify the subset of studies that met the criteria listed in step 2 (above). We also scanned reference lists from relevant reviews.^11^, ^12^, ^13^, ^20^, ^21^, ^22^

### 2.4 Step 4: Charting the data

Two reviewers independently extracted data from the included studies. We summarized studies according to study characteristics and reported a narrative synthesis of the results.^15^, ^16^ In keeping with scoping review methodology,^17^ we did not undertake a formal quality assessment of the studies.

### 2.5 Step 5: Collating, summarizing, and reporting the results

We reviewed the results in an iterative manner, suggested refinements, and provided insights on the findings.

### 2.6 Step 6: Consulting with stakeholders

Members of the study team include representatives from organizations that represent nursing home staff, families, and residents. These stakeholders became involved in the review after the publication of the study protocol.^14^ The first reports highlighted mental health outcomes and potential strategies during COVID‐19.^18^, ^23^ The second publication focused on physical health outcomes.^24^ This, the third and final publication, specifically examined the impact of nursing home and community characteristics on social connection and how this phenomenon is being measured in the literature. This publication stemmed from the stakeholder's expressed desire to synthesize evidence with eventual implications for policy and planning. They helped define the review questions, interpreted and contextualized results and coauthored publications.

## 3 RESULTS

**Figure 1:**
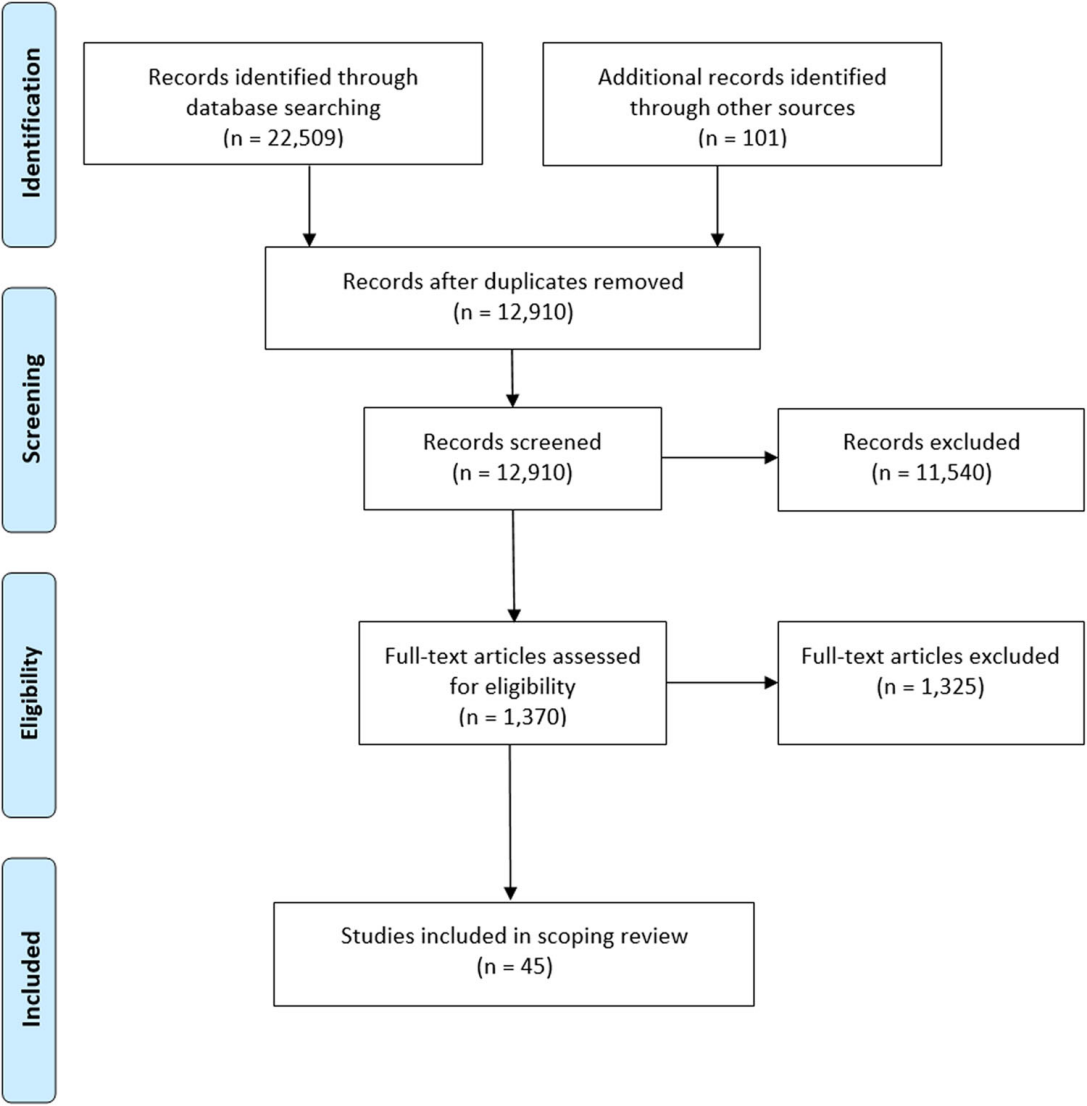
PRISMA flow diagram

The search strategy yielded 22,509 titles, which reduced to 12,910 after deduplication and searching reference lists. After screening and full‐text review, 45 papers remained (see Figure 1). Characteristics of the included studies are summarized in Tables 1 and 2. See Supporting Information: Appendix B for detailed descriptions. Most studies (*n* = 27; 60%) were published since 2010, conducted in North America (*n* = 24; 53%), used a cross‐sectional or pre‐post study design (both *n* = 16; 36%) and had a sample size of 100–249 (*n* = 16; 36%) residents and less than 10 homes (*n* = 20; 44%). The most frequently reported interventions/exposures assessed small‐scale home‐like settings (*n* = 17; 38%), facility characteristics (*n* = 14; 31%); staffing (*n* = 11; 24%), care philosophies (*n* = 9; 20%), and community characteristics (*n* = 5; 11%). All studies assessed nursing home characteristics and a subset of five studies also assessed the impact of community characteristics. Eight studies assessed multiple home or community level exposures. Studies most often created indicators or counts of social activities, visits, or contacts with residents and staff (*n* = 11; 24%) as outcome measures to assess social connection. Eleven distinct aspects of social connection were reported. The most common scales used to assess social connection were those devised for health administrative data and use with all nursing home residents (i.e., Index of Social Engagement^25^ (*n* = 8; 18%) and Revised Index of Social Engagement^26^ (*n* = 4; 9%) or the social relations (*n* = 6; 13%) and social isolation (*n* = 5; 11%) subscales of QUALIDEM,^27^ a quality of life measure developed for persons with dementia. Ten studies assessed multiple social connection outcomes. Most studies (*n* = 36; 80%) did not measure social connection using resident's self‐reported information.

**Table 1:** Description of published research articles included in scoping review

Study characteristics	TOTAL (*n* = 45)	
	*N*	%
Year of publication		
Pre‐1990	2	4.4
1990–1999	5	11.1
2000–2009	11	24.4
2010–2020	27	60.0
Region		
Asia	2	4.4
Europe	17	37.8
North America (United States)	24 (20)	53.3 (44.4)
Other/multiple	2	4.4
Study design		
Cross‐sectional	16	35.6
Pre–Post	16	35.6
Cohort	10	22.2
Randomized controlled trial	1	2.2
Other/not stated	2	4.4
Sample size (nursing home residents)		
Less than 100	13	28.9
100–249	16	35.6
250–499	6	13.3
500 or more	9	20.0
Not stated	1	2.2
Sample size (nursing homes)		
Less than 10	20	44.4
10–49	12	26.7
50–99	2	4.4
100–249	5	11.1
250+	3	6.7
Other (wards/multiple types of homes)	3	6.7
Resident‐reported measures of social connection		
Yes	9	20.0
No	36	80.0

**Table 2:** Description of social connection outcomes in studies reviewed

Measure	TOTAL (*n* = 45)	
	*N*	%
Index of social engagement	8	18
Qualidem, social relations subscale	6	13
Qualidem, social isolation subscale	5	11
Revised index of social engagement	4	9
Multidimensional observation scale for elderly subjects (MOSES), social withdrawal subscale	4	9
Quality of life instrument, relationships subscale	2	4
Proposed minimum data set (MDS) 3.0 section F, relationships	2	4
Assessment tool for occupation and social engagement (ATOSE)	2	4
UCLA loneliness scale	1	2
Maastricht electronic observation tool (MEDLO‐tool), social interaction	1	2
QUALID, social interaction subscale	1	2
Inventory of socially supportive behaviors	1	2
Social well‐being of nursing home residents (SWON)	1	2
Sanson‐fisher behavioral mapping, group behaviors	1	2
Interview schedule for social interaction (ISSI)	1	2
Bennett's past month index, social network	1	2
Resident assessment instrument minimum data set (RAI‐MDS), activity pursuit patterns characterized as interaction with others	1	2
Social network, concentric circle approach	1	2
**Study‐specific measures**:		
Social engagement, using indicators or counts of participation in various social activities and contacts with visitors, staff, and residents	11	24
Social interaction, using direct observation	3	7
Social support, measure of inclusion of informal caregivers in nursing and care	1	2
Loneliness, single‐item questions	1	2

*Note*: Column percent adds to more than 100% because some studies investigated multiple aspects of social connection.

### 3.1 Home‐level: Facility

Fourteen studies assessed various facility characteristics, including attributes of the building, environment and resident population, and most of the studies assessed multiple exposures.

#### 3.1.1 Home size (i.e., number of beds or residents)

Six studies testing the association between home size and residents' social connection produced mixed results. Three studies found the smaller home size was associated with better social connection, including higher social engagement,^28^, ^30^ and less social isolation.^30^ Conversely, one study found no statistically significant association between home size and relationships^31^ and other studies reported larger home size was associated with higher social engagement,^32^ and reduced social withdrawal.^33^ Some studies analyzed a continuous variable^29^, ^32^, ^33^ and others used different characterizations of “small”, “medium” and “large”^28^, ^30^, ^31^; for the latter, “large” homes were those with more than 100 residents.

#### 3.1.2 Ownership (i.e., profit status)

Five studies tested the association between ownership and residents' social connection, and none reported a statistically significant association.^28^, ^29^, ^31^, ^32^, ^33^

#### 3.1.3 Type of ward/unit (i.e., dementia care unit)

Five studies examined the type of ward or unit within the home and produced mixed results. Two studies reported a significantly higher social connection among residents in dementia special care units, in particular higher social engagement^34^ and social contact with staff.^35^ A third study found that residents in dementia special care units were more likely to have social interactions, but only in the afternoon.^36^ Conversely, one study reported no association between residing in a special care unit and social engagement^32^ and another study found that among new nursing home residents, the bivariate association between ward type and social engagement disappeared in regression models, attributing the differences between wards to resident levels of depression and functional and cognitive impairments.^37^

#### 3.1.4 Shared rooms/privacy

Two studies tested the association between measures of shared rooms and residents' social connection and neither reported a statistically significant association. The proportion of private rooms within the nursing home was not associated with relationship scores^31^ and type of room (single or shared) did not predict social engagement.^34^

#### 3.1.5 Environment

Two studies each assessed multiple aspects of home and building environment. One study assessed temperature, noise and lighting level in different areas of the home (living room, bedroom, and dining room) among residents with advanced dementia; only the association between high noise levels in the living room and less social interaction was statistically significant in adjusted models.^38^ Another study, conducted in dementia special care units, tested ratings of exit control, walking paths, individual space, common space, outdoor freedom, residential character, autonomy support and sensory comprehension; only the association between increasing common spaces variability (i.e., uniqueness) and decreasing social withdrawal was statistically significant.^33^

#### 3.1.6 Medicaid census

Two studies reported on the association between Medicaid census (i.e., proportion of residents on Medicaid) and residents' social connection. One found higher Medicaid census was associated with improvement in social engagement score^32^ whereas the other did not.^39^

#### 3.1.7 Other facility characteristics

Four studies reported on other aspects of the home. One study reported occupancy rate and chain affiliation were not associated with social engagement.^32^ A study that tested the association between dementia friendliness of the nursing home's mission statement and social withdrawal did not report a statistically significant association.^33^ Another study assessed the influence of social capital, defined as collective norms of trust and reciprocity, within nursing homes; it found social capital influenced residents' mental and functional health, but contrary to hypothesis, the effect was not through social support and social engagement.^40^ Presence of a resident dog initially increased interactive behaviors for both staff and residents, however, by 22 weeks, resident behaviors had reverted to baseline levels.^41^

### 3.2 Home‐level: Care philosophy

Nine studies examined the impact of seven care philosophies on aspects of social connection but only three reported statistically significant results.

#### 3.2.1 Restorative care

Two studies assessed restorative care, which emphasizes maintaining, restoring and optimizing residents' function, and one reported statistically significant results. The first study, assessing social support at baseline and 6 months after implementing a restorative care intervention, reported significant improvements in social support overall and for emotional and informational support domains.^42^ The second study, testing the effect of a restorative care training and education program for supervisory and direct care staff, and comparing to usual care, suggested improvements in residents' social withdrawal, but the results were not statistically significant.^43^

#### 3.2.2 Eden alternative

Two studies assessed the Eden Alternative, which emphasizes resident interactions with plants, animals, and children for quality of life, and neither reported statistically significant results. The first study, conducted in cognitively intact older adults from a state veterans home, reported no change in residents' loneliness.^44^ The second study, comparing data from residents living in a nursing home before and after the Eden Alternative was implemented, also reported no statistically significant change in social engagement.^45^

#### 3.2.3 Other care philosophies

Five additional studies assessed care philosophies and two reported statistically significant findings. One observational study assessed the influence of culture change, measured with a survey of administrators and social service directors that assessed aspects of resident care, nursing home environment, relationships, staff empowerment, nursing home leadership, shared values, and quality improvement. It found the relationship subscale of culture change positively predicted residents' social networks.^40^ One quasi‐experimental study assessed the impact of the Veder contact method, a person‐centered method using theatrical, poetic and musical communication for care; compared to care as usual, the Veder contact method group showed positive improvements in the residents' social relations but not social isolation.^46^ A quasi‐experimental study testing the Quality of Life Nursing Care model, focusing on choice and control, consistent staff assignments, case‐management and resident scheduling, included social network, social engagement and loneliness outcomes, but reported no statistically significant findings.^47^ A randomized trial, conducted in residents with dementia and testing the impact of integrated emotion‐oriented care versus usual care, did not find a statistically significant difference in residents' social relationships.^48^ A quasi‐experimental study evaluating a person‐centered care program (P.I.E.C.E.S.™) reported the intervention had no effect on resident social engagement.^49^

### 3.3 Home‐level: Small‐scale, home‐like setting

Seventeen studies examined homes that combined small‐scale (small units vs. larger traditional units) with a home‐like setting, with most exclusively studying residents with dementia.

#### 3.3.1 *Green care farms* (i.e., homes that combine agricultural with care activities)

Two related studies suggested better social connection compared to traditional nursing homes, but not compared with regular small‐scale living facilities.^50^, ^51^ More specifically, compared to residents of traditional nursing homes, cross‐sectional analysis showed that those in Green Care Farms scored higher on social relations, but not social engagement or social isolation^50^ and at 6‐month follow‐up, residents of Green Care Farms had significantly more social interaction.^51^

#### 3.3.2 *Green house homes* (i.e., an approach to nursing home building, environmental design, daily life, and care as well as staff and resident roles)

Two studies assessed Green House Homes with both suggesting better social connection outcomes. One quasi‐experimental study found that relationships scores were higher for residents of Green House Homes than in residents in the rest of the nursing home but the difference with another nearby nursing home was not statistically significant.^52^ Another cohort study found that, compared to traditional nursing home residents, the trajectory of social engagement over time may be better for Green House Home residents.^53^

#### 3.3.3 *Dementia special care units* (i.e., small‐scale home‐like settings in dementia special care units)

Seven studies assessed dementia special care units and most did not report statistically significant results. One study found social interaction was significantly related to this type of setting^54^ and one found that social interaction was weakly related to group living characteristics.^55^ However, five studies produced results that were not statistically significant.^56^, ^57^, ^58^, ^59^, ^60^

#### 3.3.4 Other household/group‐living models

Six studies assessed other small‐scale settings which suggested potential benefit. One cross‐sectional study of residents (with or without dementia) found that residents and staff spent more time engaged in social interactions with the household model compared to traditional nursing homes.^61^ Two papers that reported from the same data of residents with dementia, pre‐ and post‐conversion from a traditional to a household model unit, found social engagement increased post‐conversion.^62^, ^63^ Another study of residents with dementia in group‐living home‐like settings found social engagement was higher among residents of group‐living homes compared to traditional nursing home settings.^64^ One longitudinal study of residents with dementia found that although social connection was higher in small‐scale home‐like settings, the difference was not sustained over time.^65^

Another longitudinal study of residents of nursing homes in Belgium and the Netherlands reported that, in the Dutch homes, residents in small‐scale settings had higher mean scores on social relations but there were no differences for social isolation or social engagement (as assessed with the Revised Index of Social Engagement and number of visits).^66^

### 3.4 Home‐level: Staffing

Eleven studies assessed staffing characteristics, with most studying attributes of nursing care staff.

#### 3.4.1 Staffing level, mix, and staff‐to‐resident ratios

Six studies tested the association between staffing level, skill mix or staff‐to‐resident ratios, and social connection. Three of these studies reported significant results, but only for certain staff categories. The first study tested each ratio of registered nurses (RN), licensed practical nurses (LPNs), and nurse aides to residents and found lower LPNs per resident and higher nurse aides per resident were associated with improvements in social engagement.^32^ The second study found that higher staffing levels of personal care assistants were associated with higher social engagement.^28^ The third found relationships were negatively related to RN staffing hours.^67^ A fourth study found RN to certified nursing assistant ratio was not significantly related to social engagement.^29^ Two studies reported no statistically significant associations; one tested the association between total staff to resident ratios and social withdrawal,^33^ and another tested the relationship between hours per resident day for RN, LPN, and certified nursing assistant, as well as skill mix and turnover for each category of staff.^68^

#### 3.4.2 Nurse aide job characteristics

Two studies examined specific aspects of the roles of nurse aides. One study collected information about nurse aides' stability (turnover and retention), empowerment strategies (e.g., delegation, influence over resident care decisions), registered nurse‐to‐nurse aide ratio and nurse aide unionization, to study their impact on resident social engagement. They found the amount of influence nurse aides had in resident care decisions predicted residents' social engagement, and social engagement scores were lower in facilities experiencing either high turnover and low retention or low turnover and high retention relative to facilities where both turnover and retention were high.^29^ Another study evaluated the impact of a primary care nursing model on social interactions, using a permanent assignment of nursing aides to residents, a “teams‐of‐two” approach and enhanced communication between aides and other staff. This study found social interactions were positively associated with the use of this model of care.^69^

#### 3.4.3 Other aspects of staffing

Three other studies examined aspects of staffing, including the impact of specific roles (social workers and a geriatric nurse practitioner) and staff attitudes. A multi‐level cross‐sectional study found that living in nursing homes with greater numbers of social workers was positively associated with social support.^40^ A cohort study suggested the presence of a geriatric nurse practitioner did not improve social interaction among residents.^70^ A cross‐sectional study assessed the association between staff attitudes towards dementia and residents' social well‐being; results showed that when care staff had a more hopeful attitude towards residents with dementia, residents displayed higher social well‐being, but there was no statistically significant association for the “person‐centeredness” attitude subscale.^71^

### 3.5 Community‐level

Five studies tested a range of community characteristics. Typically measures described the population in the area surrounding the home and were defined from census data. The first considered county‐level unemployment rates but was not significantly related to social engagement.^29^ The second reported higher levels of market competition (Herfindahl Index); lower numbers of older adults and higher average incomes in the county were all associated with improvement in social engagement.^32^ Another study used measures of the proportion of the Census tract community by race and ethnicity group, working class, urban category, education, age, home value, and poverty ranking; the only statistically significant finding linked location in urban communities to lower social engagement.^39^ Three other studies also reported results testing the association between rural versus urban home locations and social connection; one found nursing homes in urban areas had higher levels of social engagement^28^ and the other two found no significant association with relationships scores^31^ or social withdrawal.^33^

## 4 DISCUSSION

Our scoping review included 45 studies that assessed home‐ and community‐level predictors of social connection in nursing home residents. All 45 studies examined home‐level characteristics whereas only five also analyzed community‐level characteristics. The studies reported 11 distinct aspects of social connection and 22 approaches to measurement. Overall, findings were mixed, however potentially promising results were found in studies examining the impact of small‐scale home‐like settings. Our scoping review highlights knowledge gaps and points to the need for research that will inform policy, care planning, and evaluation.

This study builds on previous reviews^11^, ^12^, ^13^, ^20^, ^21^, ^22^ by focusing on literature that quantifies distinct aspects of social connection and includes community‐level characteristics as well as summarizing approaches to measuring social connection in nursing homes. We found the number of studies in this area has increased substantially since Xu et al's review. Similar to Brownie and Nancarrow's review of person‐centered care, we found the majority of the intervention studies used quasi‐experimental designs rather than randomized controlled trials. Like Armijo‐Olivo et al's review of nursing staff time and quality of care and quality of life, we found mixed results from studies of staffing, including RN skill mix, which may be explained by the more clinical nature of RN roles and responsibilities. As with Chaudhury et al.'s and Ferdous' reviews, we found studies that linked the physical design of nursing home environments to social connection, however, we found these studies sometimes also incorporated a care philosophy. Adlbrecht et al's review addressed the relationship between physical design and social connection in special care units, similarly concluding that despite a weak evidence base, these settings can have a positive impact on residents.

Taken together, these reviews highlight an evolving evidence base and point to the need for carefully designed prospective observational and intervention studies that test the impact of strategies delivered at the home and community level. In particular, the findings point to a range of potentially modifiable characteristics where research would have important implications for policy and practice at the home and health system levels. For example, at the health system level, evidence in this area would help to guide decisions about where and how to build new homes as well as for determining optimal staffing levels and mix. At the home level, our findings point to potential strategies related to the built environment, care philosophy, human resources (e.g., training, hiring, and retention), communication and staff roles and responsibilities within the home. Other innovations, such as incorporating creative art^72^ and technology installations,^73^ although not included in our review, may also present promising approaches. While our review was initiated before the COVID‐19 pandemic and the included studies did not occur or discuss their findings in the context of pandemics or infectious disease outbreaks, COVID‐19 highlighted social connection as an important public health issue for nursing homes. Despite this limited evidence base, work to establish standards for nursing homes in the wake of COVID‐19,^74^, ^75^ including to address infection control and emergency and disaster preparedness plans, must equally address the imperative of building and maintaining social connection for residents.

Several specific knowledge gaps also emerged during consultation with stakeholders involved in this review. First, studies of staffing were limited. They focused almost exclusively on nursing staff and care aides and none tested the impact of therapeutic recreation, dietary and cleaning staff, volunteers, or other external services. Staffing studies mainly addressed staffing level or skill mix while very few collected more detailed information about job characteristics^29^ or staff attitudes.^71^ Despite the crucial roles of leadership (e.g., administrators and directors of care/nursing) in influencing quality of care,^76^ we found no studies testing these effects. Second, there were also no studies that assessed the impact of culturally‐^77^ or ethno‐specific^78^ nursing homes where care is tailored to a particular group, including through aspects such as staffing, offering specific foods and activities as well as building design.^79^ Third, we found only five studies of community‐level characteristics and most used census‐derived measures of the population surrounding the home. A report of case studies from LTC homes in Canada, Norway, and Germany found that families and residents highlighted the importance of neighborhood amenities that allow residents to engage with the community and visitors.^8^, ^80^ Yet, to our knowledge, no quantitative studies have corroborated the association between the built environment surrounding the LTC home and residents' social connection. Fourth, over half of the studies identified in this review were observational and we found only one randomized controlled trial; while we intentionally included both observational and interventional studies, this finding supports others' calls for strategies to address the challenges in conducting clinical trials in nursing homes.^81^, ^82^ Finally, the multiple approaches to measurement suggest a lack of consensus that may extend more broadly to research on social connection in this population.

To our knowledge, this study is the first to focus on home‐level and community‐level predictors of social connection in nursing home residents. However, we acknowledge several limitations in our study. First, our findings are limited by an inexhaustive review of the literature; only English language studies were included^18^ and, despite a thorough search strategy, some relevant studies may have been missed. Second, our scoping review was broadly inclusive, which limited our interpretation of study findings in the context of nursing home populations and systems that have changed over time and differ between countries. Third, we presented nursing home and community‐level characteristics as reported in the studies, however, we acknowledge ambiguity in some of these concepts may have obscured differences within categories. Finally, while measures have been tested in nursing home residents,^25^, ^26^, ^27^, ^83^ it was beyond the scope of the current review to assess the quality of the evidence for these instruments and, acknowledging issues of inconsistent terminology in this area of research,^84^ the aspect(s) of social connection each measure assessed.

In conclusion, we found research assessing small‐scale home‐like settings, facility characteristics, staffing characteristics, care philosophy, and community characteristics as predictors of social connection in nursing home residents**.** The increasing number of studies in this area likely reflects an increasing recognition of the importance of the topic^85^; that is, social connection is an important aspect of quality of life and care in nursing homes, as well as a predictor of good physical and mental health, and strategies to address social connection should not be limited to individually based interventions.^86^, ^87^ Research testing the impact of modifiable home‐ and community‐level factors on social connection should inform public policy and local planning^88^ when, for example, building and designing nursing homes as well as resourcing and delivering care. Further research is needed to build an evidence‐base in this area, including to address measurement issues and to study the impact of built environment, staffing and care philosophy characteristics and the interactions between these factors.

## AUTHOR CONTRIBUTIONS

*Conceptualization*: Katherine McGilton, Andrea Iaboni, Jennifer Bethell, Katelynn Aelick, Jessica Babineau, Monica Bretzlaff, Cathleen Edwards, Josie‐Lee Gibson, Debbie Hewitt Colborne, Dee Lender, Denise Schon, and Ellen Snowball. *Formal analysis*: Katherine McGilton, Jennifer Bethell, Sara Clemens, Katelynn Aelick, Jessica Babineau, Monica Bretzlaff, Cathleen Edwards, Josie‐Lee Gibson, Debbie Hewitt Colborne, Dee Lender, Denise Schon, and Ellen Snowball. *Funding acquisition*: Jennifer Bethell. *Writing*—*review and editing*: Katherine McGilton, Andrea Iaboni, Jennifer Bethell, Sara Clemens, Katelynn Aelick, Jessica Babineau, Monica Bretzlaff, Cathleen Edwards, Josie‐Lee Gibson, Debbie Hewitt Colborne, Dee Lender, Denise Schon, and Ellen Snowball. *Writing*—*original draft*: Jennifer Bethell, Sara Clemens. All authors have read and approved the final version of the manuscript. As lead author, Dr. Sara Clemens had full access to all of the data in this study and takes complete responsibility for the integrity of the data and the accuracy of the data analysis.

## CONFLICT OF INTEREST

The authors declare no conflict of interest.

## ETHICS STATEMENT

The manuscript submitted to *Health Science Reports* has been done in accordance to “Wiley's Best Practice Guidelines on Publishing Ethics” and has been performed in an ethical and responsible way, with no research misconduct, which includes, but is not limited to data fabrication and falsification, plagiarism, image manipulation, unethical research, biased reporting, authorship abuse, redundant or duplicate publication, and undeclared conflicts of interest.

## TRANSPARENCY STATEMENT

As lead author, Dr. Sara Clemens affirms that this manuscript is an honest, accurate, and transparent account of the study being reported; that no important aspects of the study have been omitted; and that any discrepancies from the study as planned (and, if relevant, registered) have been explained.

## Supporting information

Supporting information.

## Data Availability

All relevant data are included in the review and/or its supplementary information files.
