# Supplementary material for: Home‐ and community‐level predictors of social connection in nursing home residents: A scoping review
Source: Health Sci Rep. 2022 Jul 20;5(4):e743. doi: 10.1002/hsr2.743 (PMC9297378; doi:10.1002/hsr2.743)
Supplement: Supplementary file 1 — Supporting information. [file HSR2-5-e743-s001.docx]

# Supplementary Material – Appendix A: Search Strategy

**Search Narrative**

This strategy was first developed in Medline and was then translated to other databases. It uses a number of concepts:

Concept A: lines 1 to 38 = Social engagement, social support, social capital, loneliness or social isolation

Concept B: lines 39 to 58 = Long term care, or nursing homes, homes for the aged

The search conducted is: (A AND B)

This strategy was used in all searched databases. Searches were also limited to adult human populations, and English language when possible.

In other databases, additional limits and search fields have been used when applicable such as to exclude conference proceedings.

**Medline (Ovid) Search Strategy**

1 Interpersonal Relations/

2 exp social support/

3 Social Participation/

4 exp Social Isolation/

5 Social inclusion/

6 Social Interaction/

7 Social Network Analysis/

8 Loneliness/

9 Social Behavior/

10 ((social or socially) adj3 (engaged or engagement)).tw,kf.

11 ((social or socially) adj3 (disengaged or disengagement)).tw,kf.

12 ((social or socially) adj3 (connect* or disconnect*)).tw,kf.

13 Sociali?ation.tw,kf.

14 Sociali??.tw,kf.

15 sociali?ing.tw,kf.

16 Interpersonal relation?.tw,kf.

17 (social adj3 behavio?r*).tw,kf.

18 (social adj3 capital?).tw,kf.

19 ((social or socially) adj3 support??).tw,kf.

20 (social adj3 relationship?).tw,kf.

21 ((social or socially) adj3 participat*).tw,kf.

22 (social adj3 network?).tw,kf.

23 (social adj3 interaction*).tw,kf.

24 (personal adj3 network?).tw,kf.

25 friendship?.tw,kf.

26 ((social or socially) adj3 integrat*).tw,kf.

27 (social adj3 relation?).tw,kf.

28 (social adj3 contact).tw,kf.

29 ((social or socially) adj3 embedded*).tw,kf.

30 lonel*.tw,kf.

31 ((social or socially) adj3 isolat*).tw,kf.

32 (emotional* adj3 isolat*).tw,kf.

33 ((perceive* or percept*) adj3 isolat*).tw,kf.

34 ((social* or socially) adj3 alienat*).tw,kf.

35 aloneness.tw,kf.

36 solitud*.tw,kf.

37 Unwanted alone.tw,kf.

38 or/1-37

39 Nursing Homes/

40 Residential Facilities/

41 Homes for the Aged/

42 Long-Term Care/

43 "long-term care".tw,kf.

44 "longterm care".tw,kf.

45 LTC.tw,kf.

46 ((home? or facility or facilities) adj2 (retirement or assisted living or residential care or extended care)).tw,kf.

47 home? for the aged.tw,kf.

48 home? for the elderly.tw,kf.

49 old age home?.tw,kf.

50 ("old age facility" or "old age facilities").tw,kf.

51 (nursing adj3 (facility or facilities or residence? or center? or centre?)).tw,kf.

52 nursing home?.tw,kf.

53 (residence? adj2 ('assisted living' or convalescen* or retire???? or 'long stay' or longstay or 'long term')).tw,kf.

54 ((facility or facilities) adj2 ('assisted living' or convalescen* or retire???? or resident* or 'long stay' or longstay or 'long term')).tw,kf.

55 (home? adj2 ('assisted living' or convalescen* or retire???? or 'long stay' or longstay or 'long term')).tw,kf.

56 "care home?".tw,kf.

57 (housing adj3 (retirement or old age or senior* or elderly)).tw,kf.

58 or/39-57

59 38 and 58

60 59 not ((exp infant/ or exp child/) not exp adult/)

61 60 not (exp animals/ not exp humans/)

62 limit 61 to english language

# Supplementary Material – Appendix B: Study Descriptions

| **First Author** | **Title** | **Year** | **Country** | **Population** | **No. residents (No. homes)** | **Incl/excl for Cognition** | **Study design** | **Home / Community Characteristics** | **Outcome Measured** | **Findings** |
| --- | --- | --- | --- | --- | --- | --- | --- | --- | --- | --- |
| Curry | The Effects of Nursing Home Size on Resident Isolation and Life Satisfaction | 1973 | United States | Nursing home residents | 200 (26) | All were mentally competent | Cross-sectional | Nursing home size categorized as large (over 100 beds), intermediate-sized (50-99 beds) and 28 small (up to 49 beds) | Social isolation | Only 5% of the residents from the smaller homes were totally isolated from contact with friends or relatives, compared with 23% and 22% for intermediate-sized and large homes, respectively (p<0.01). Residents of the smaller homes have more friends within the home (p<0.05) and more monthly contacts with those friends within the home (p<0.0l). Residents of the intermediate and larger homes have more monthly contact with relatives (p<0.0l). |
| Winkler | The Impact of a Resident Dog on an Institution for the Elderly: Effects on Perceptions and Social Interactions | 1989 | Australia | Nursing home residents | 20 (1) | None stated | Pre-/post- | Dog therapy | Solitary, dyadic communication, and group behaviors | Six weeks after the dog's arrival, a significant increase in frequency of interactive behaviors was seen for both staff and patients. By 22 weeks, behaviors for patients had reverted to base-line levels, while remaining high for staff. |
| Garrard | Impact of Geriatric Nurse Practitioners on Nursing-Home Residents' Functional Status, Satisfaction, and Discharge Outcomes | 1990 | United States | Nursing home residents, new residents and long-stay | 525 (323) | None stated | Cohort | Geriatric nurse practitioner (GNP) (vs comparison homes without a nurse practitioner) | Social interaction | Among new residents, the non-GNP group showed greater improvement in social interaction scores than did the GNP group. |
| Cox | Quality of Life Nursing Care: An Experimental Trial in Long-Term Care | 1991 | United States | Nursing home residents | 45 (1) | Inclusion: included only those who were cognitively intact | Pre-/post- | Quality of Life Nursing Care model (vs. control) | Social network, loneliness | No statistically significant differences reported. |
| Teresi | A Primary Care Nursing Model in Long-Term Care Facilities: Evaluation of Impact on Affect, Behavior, and Socialization | 1993 | United States | Long-term care residents in urban and rural homes | 260 (2) | None stated | Pre-/post- | Primary care model of delivery of nursing aide care (vs comparison), using permanent assignment of nursing aides, a teams-of-two approach and enhanced communication between aides and other staff. | Social interaction | In the rural experimental home, there were significant improvements in social activities (interaction) in the experimental group (p=0.01). At the urban home, there was no impact on social engagement. |
| Kovach | Impacts of a therapeutic environment for dementia care | 1997 | United States | Nursing home residents | 12 (2) | Inclusion: dementia | Cohort | Pre and post relocation from a traditional nursing home to a dementia care facility | Social interaction | There was a significant increase in observed social interaction between the two settings (p<0.005); more social interaction was observed in the dementia care unit. |
| Castle | Organizational Structure and Outcomes for Nursing Home Residents with Mental Disorders | 1998 | United States | Nursing home residents with mental disorders (n= 2,170 in 1990 and n=2,088 in 1993) | 2,170 in 1990 and 2,088 in 1993 (268) | Residents had mental disorder | Cohort | Home-level: staffing levels (FTEs of nurse aides, licensed practical nurses (LPNs), and registered nurses (RNs) per resident); size (number of beds); chain (member of a nursing home chain or not); for-profit or not-for-profit; occupancy rate (average occupancy rate); Medicaid (average Medicaid occupancy rate); special care units (Alzheimer’s special care unit or not). Community-level: munificence (a) the number of elderly in the county, (b) average income in the county, and c) the number of hospital beds per 10,000 population | Social engagement | The variables associated with improved social engagement were: lower FTE LPNs per resident (p<0.01), higher FTE nurse aides per resident (p<0.01), larger size (p<0.01), higher levels of market competition (p<0.01), higher average incomes in the county (p<0.01), higher average incomes in the county (p<0.01), retrospective Medicaid reimbursement (p<0.001), higher Medicaid census and number of elderly in the county (p<0.10). Alzeimer unit was not sig rel to social engagement |
| Hinman | Influence of the Eden Alternative™ on the Functional Status of Nursing Home Residents | 2002 | United States | Assisted living and long-term care facility residents | 19 (1) | None stated | Pre-/post- | Pre and post implementation of Eden Alternative | Social engagement | No statistically significant differences reported. |
| Achterberg | The Effect of Depression on Social Engagement in Newly Admitted Dutch Nursing Home Residents | 2003 | The Netherlands | Newly admitted nursing home residents | 562 (65) | None stated | Cross-sectional | Ward type (somatic, psychogeriatric, or rehabilitation) | Social engagement | The bivariate correlation between ward types and social engagement disappeared in adjusted multivariate logistic regression. |
| Zeisel | Environmental Correlates to Behavioral Health Outcomes in Alzheimer’s Special Care Units | 2003 | United States | Nursing home special care unit residents | 427 (15) | Inclusion: Alzheimer’s disease or a related disorder | Cross-sectional | Environmental design features: exit control, walking paths, individual space, common space, outdoor freedom, residential character, autonomy support and sensory comprehension. Facility-level characteristics: staff/resident ratio, facility size, organization (not-for-profit or for-profit status), and degree of dementia friendliness, rurality | Social withdrawal | The amount of variability among common spaces was inversely correlated with social withdrawal; social withdrawal decreased as the variability among the common spaces increased. Larger facilities were associated with lower social withdrawal. |
| Bergman-Evans | Beyond the Basics: Effects of the Eden Alternative Model on Quality of Life Issues | 2004 | United States | Nursing home residents | 34 (2) | Inclusion: cognitively intact | Pre-/post- | Eden Alternative model (vs private long-term care facility) | Loneliness | There was no change in the level of loneliness from baseline to follow up for the Eden group and no difference with the control group (p=0.34). |
| Kane | Using Resident Reports of Quality of Life to Distinguish Among Nursing Homes | 2004 | United States | Nursing home residents | 2000 (40) | 50/50 split in those with high and low cognitive scores | Ecological | Facility characteristics: size, urban vs. rural, not-for-profit vs. for-profit, % private rooms, number of beds (categorized as small (fewer than 109) or large (109 or more)) | Relationships | No statistically significant associations between facility characteristics and relationships scores |
| Reimer | Special Care Facility Compared with Traditional Environments for Dementia Care: A Longitudinal Study of Quality of Life | 2004 | Canada | Long-term care and assisted living residents | 185 (1) | Inclusion: middle-to late-stage dementia (score of 5 or more on the Global Deterioration Scale) | Cohort | Specialized care facility (vs. traditional institutional facilities) | Social withdrawal | There were no differences between groups in social withdrawal. |
| Barry | Nurse Aide Empowerment Strategies and Staff Stability: Effects on Nursing Home Resident Outcomes | 2005 | United States | Nursing home residents | Not stated (156) | None stated | Cross-sectional | Home-level characteristics: nurse aide empowerment strategies and staff. stability (turnover, i.e., number of nurse aide resignations or terminations relative to the number of nurse aides employed in the facility for the 6 months; retention, i.e., proportion of nurse aides employed at the facility for 2 years or longer; delegation; influence; rewards; advanced nurse aide position; nurse aides serve on committees; and facility uses job enhancement techniques), home size (total number of beds), profit status (for profit or not for profit), registered nurse-to-nurse aide ratio (registered nurse full-time equivalents, or FTEs, to nurse aide FTEs) and nurse aide unionization. Community-level: county-level unemployment rate | Social engagement | Facility size was inversely associated with social engagement (p<0.05), amount of influence nurse aides have in resident care decisions was positively associated with social engagement (p<0.001). Social engagement scores were lower in homes with either high turnover + low retention (p<0.05) or low turnover + high retention (p<0.10), compared to homes where both turnover and retention were high). Low turnover + low retention not statistically significant. |
| Finnema | The effect of integrated emotion-oriented care vs usual care on elderly persons with dementia in the nursing home and on nursing assistants: a randomized clinical trial | 2005 | The Netherlands | Nursing home residents | 146 (58) | Inclusion: probable diagnosis dementia of the Alzheimer, Type (DAT), combined DAT and vascular dementia, dementia syndrome (NAO) or amnestic syndrome | Randomized clinical trial | Integrated emotion-oriented care (vs usual care) | Social relationships | No significant differences between the groups in social relationships. |
| Johnson | Evaluation of the Restorative Care Education and Training Program for Nursing Homes | 2005 | Canada | Nursing home residents | 84 (12) | None stated | Pre-/post- | Restorative care (vs usual care) | Social withdrawal | Trends towards improvement in the intervention group and decline in the control, but difference was not statistically significant (p=0.21). |
| Kane | Resident Outcomes in Small-House Nursing Homes: A Longitudinal Evaluation of the Initial Green House Program | 2007 | United States | Nursing home residents | 1316 (4) | None stated | Pre-/post-; SC: "longitudinal quasi-experiment" | Green house home (vs sponsoring nursing home and traditional nursing home) | Social engagement and Relationships | The Green House group were less likely to participate in organized activities in the facility, but more likely to participate in organized trips away from the home. The Green House group was just as likely to engage in solo activities, receive phone calls and visits, take privately arranged trips from the setting, or have an overnight guest. Green House residents reported better relationships scores than one of the comparison nursing homes (p=0.002). |
| Te Boekhorst | The Effects of Group Living Homes on Older People with Dementia: A Comparison with Traditional Nursing Home Care | 2009 | The Netherlands | Nursing home residents | 164 (104) | Inclusion: dementia | Pre-/post- | Group living homes (vs traditional nursing homes) | Social engagement and Social relations | Social engagement was higher among residents of group living homes (p<0.05) |
| McFadden | Continuity in the midst of change: behaviors of residents relocated from a nursing home environment to small households | 2010 | United States | nursing home residents | 22 (2) | Exclusion: residents in the acute terminal phase of life and residents at risk for wandering | Pre-post- | Newly constructed smaller house-hold units, designed for residents with dementia | Social interaction | No significant differences before and after relocation, with the exception of nonverbal social interactions. Overall, residents showed consistency in both social and nonsocial behaviors |
| Verbeek | Dementia Care Redesigned: Effects of Small-Scale Living Facilities on Residents, Their Family Caregivers, and Staff | 2010 | The Netherlands | Nursing home residents | 259 (28 houses and 21 psychogeriatric nursing home wards | Inclusion: diagnosis of dementia according to the criteria of the Diagnostic and Statistical Manual of Mental Diseases, fourth edition | Pre-/post- | Small-scale living facilities (vs regular psychogeriatric nursing home wards) | Social isolation and social relations | No significant group by time interaction effects were found for social isolation or social relations. |
| Weyerer | Evaluation of Special and Traditional Dementia Care in Nursing Homes: Results from a Cross-sectional Study in Germany | 2010 | Germany | Nursing home residents | 1167 (39) | Inclusion: dementia (MMSE< 18) | Cross-sectional | Special dementia care units (vs traditional nursing home) | Social engagement and social support | Residents in the special care units were found to have significantly greater social contact with staff (p<0.05). |
| DeRooij | Quality of life of residents with dementia in traditional vs small-scale long-term care settings: A quasi-experimental study | 2012 | The Netherlands and Belgium | Residents of small-scale and traditional LTC homes | 179 (5) | Inclusion: dementia | Pre-/post- | Small-scale living for residents with dementia (vs traditional long-term care) | Social engagement and social isolation | For the Dutch sample, mean social engagement (RISE, p<0.01) and social relations (p<0.001) scores were higher for the small-scale facilities than for traditional setting. Over time, in the Dutch sample, mean scores on ‘social relations’ remained stable over time in traditional settings, but decreased in small-scale settings. |
| Garre Olmo | Environmental Determinants of Quality of Life in Nursing Home Residents with Severe Dementia | 2012 | Spain | Nursing home residents | 160 (8) | Inclusion: severe dementia, with Global Deterioration Scale (GDS) score of 6 points or higher | Cross-sectional | Temperature, atmospheric noise, and light level in participant’s bedroom, dining room and living room of each nursing home. | Social interaction | High noise levels in the living room were associated with low behavioral signs of social interactions. |
| Smit | The relationship between small-scale care and activity involvement of residents with dementia | 2012 | The Netherlands | Nursing home residents | 1327 (136) | None stated, but study settings for people with a primary diagnosis of dementia. | Cross-sectional | Small-scale dementia care (group living home care characteristics, and the total number of residents with dementia in the facility) | Social interaction | Residents of facilities with more group living home characteristics were more involved in social interactions (p<0.001). |
| Wolf-Ostermann | Health outcomes and quality of life of residents of shared-housing arrangements compared to residents of special care units-Results of the Berlin DeWeGE-study. | 2012 | Germany | nursing home residents (shared housing arrangement and special care units) | 56 (112) | Inclusion: dementia | Longitudinal | Shared housing arrangements compared to special care units | Social relationships / Social Isolation | No signiﬁcant differences between the two settings |
| Morgan Brown | Engaging life in two Irish nursing home units for people with dementia: Quantitative comparisons before and after implementing household environments | 2013 | Ireland | Nursing home residents with dementia | 36 (2) | Inclusion: residents with dementia | Cohort | Pre and post conversion from a traditional nursing home to a household model unit | Social engagement | Social engagement increased post conversion (p<0.001). |
| Shin | Relationship between nursing staffing and quality of life in nursing homes | 2013 | United States | Nursing home residents | 231 (25) | Exclusion: DON excluded some residents based on their cognitive status | Cross-sectional | Staffing level, skill mix and turnover (RN, LPN and CNA) | Relationships | More RN staff hours were associated with better comfort and enjoyment domains, more LPN staff hours were associated with better dignity, and more CNA staff hours were associated with better functional competence domains in this study. However, (a) as the staff hours of RNs increased, the scores of meaningful activity and relationship decreased; and (b) the contribution of LPNs/LVNs (more LPNs and LVNs to less RNs) were supported in the autonomy and spiritual well-being domains |
| Morgan Brown | Comparing communal environments using the Assessment Tool for Occupation and Social Engagement: using interactive occupation and social engagement as outcome measures | 2014 | Ireland | Nursing home residents with dementia | 36 (2) | Inclusion: residents with dementia | Cohort | Pre and post conversion from a traditional nursing home to a household model unit | Social engagement | Social engagement increased in the household model unit over the traditional model unit. |
| Shin | Nursing staffing and quality of life in Western New York nursing homes | 2014 | United States | Nursing home residents | 142 (8) | Exclusion: MMSE score of 13 or lower | Cross-sectional | Staffing numbers (head count), HPRD, age and experience (RN, LPN and CNA) | Relationships | The ratio of more RNs to fewer LPNs and CNAs had a statistically significant negative influence on the meaningfulactivity, food-enjoyment, and security domains |
| Verbeek | Effects of small-scale, home-like facilities in dementia care on residents’ behavior, and use of physical restraints and psychotropic drugs: a quasi-experimental study | 2014 | The Netherlands | Nursing home residents | 259 (28 small-scale houses and 21 traditional nursing home wards) | Inclusion: diagnosis of dementia according to the criteria of the Diagnostic and Statistical Manual of Mental Diseases, fourth edition | Pre-/post- | Small-scale living facilities (vs traditional psychogeriatric wards in nursing homes) | Social engagement | Residents in small-scale living facilities were signiﬁcantly more engaged at baseline (p < 0.001) and after six months (p = 0.038) compared with residents in traditional wards. After 12 months, no signiﬁcant differences were found. |
| Leedahl | Multilevel Examination of Facility Characteristics, Social Integration, and Health for Older Adults Living in Nursing Homes | 2015 | United States | Nursing home residents | 140 (30) | Exclusion: moderate to severe cognitive impairment (i.e., MDS 3.0 Brief Interview for Mental Status scores between 0 and 12 or MDS 2.0 Cognitive Scale scores between 3 and 10) | Cross-sectional | Culture change, rated by administrators and social service directors, with subscale scores for: resident care, nursing home environment, relationships, staff empowerment, nursing home leadership, shared values, and quality improvement. Social workers in the nursing home, experience, education, and job responsibilities. Social capital, using indicators norms of reciprocity and trust | Social network, social support, social capital | Culture change relationships sub-scale has a positive predictive relationship with social networks (p < 0.05) and greater numbers of social workers is positively associated with social support (p < 0.05). |
| Williams | An evaluation of a person-centred care programme for long-term care facilities | 2015 | Canada | Long-term care home residents | 388 (6) | None stated | Pre-/post- | Person-centred care program, P.I.E.C.E.S.™ (vs homes that had not implemented any person-centred care or similar philosophical programming before or during the study period) | Social engagement | There was no statistically significant interaction between time and condition (intervention vs. control), indicating no impact on resident social engagement. |
| Yoon | Longitudinal psychological outcomes of the small-scale nursing home model: a latent growth curve zero-inﬂated Poisson model | 2015 | United States | Nursing home residents | 242 (13) | None stated | Cohort | Green house home (vs traditional nursing home) | Social engagement | Green house home residents had a lower rate of increase of the probability of “not being socially engaged” over time compared to traditional home residents (p=0.010). |
| Lee | Effects of Physical Environment on Health and Behaviours of Residents with Dementia in Long-Term Care Facilities: A Longitudinal Study | 2016 | Canada | Nursing home residents | 12 (2) | Inclusion: early or middle stage of Alzheimer’s disease or related dementia | Cohort | Small-scale, home-like setting (vs traditional nursing home setting) | Social withdrawal | Residents in the traditional nursing home spent less time in social interaction compared to the residents in the small-scale care unit across the three assessments. But the differences in mean scores were not statistically significant for the between-subjects factor of group. |
| Yoon | Impact of the nursing home scale on residents’ social engagement in South Korea | 2016 | South Korea | Nursing home residents | 314 (10) | None stated | Cross-sectional | Nursing home size (categorized as small (10 ≤ beds < 30), medium (30 ≤ beds < 100), and large (≥ 100 beds)); location, (categorized as urban or rural); ownership (categorized as private or public); staff (ratio of residents to nursing staff and ratio of residents to personal care assistants) | Social engagement | Residents in large nursing homes had a lower probability of social engagement compared to those in medium nursing homes (OR = 0.457; p=0.005). Ratio of residents to PCA was also associated with social engagement (OR=0.585; p=0.011); higher staffing levels of PCAs associated with higher social engagement. |
| Abbott | Measuring social integration among residents in a dementia special care unit vs traditional nursing home: A pilot study | 2017 | United States | Nursing home residents | 29 (1) | Resident in dementia special care unit or medical record diagnosis of dementia (for traditional nursing home residents) | Cross-sectional | Dementia Special Care Unit vs. Traditional Nursing home | Social interaction | In the afternoon, Dementia Special Care Unit residents were more likely to have social interactions compared to the traditional nursing home residents (p=0.02). There was no difference in the morning (p=0.83). |
| Bliss | Social Engagement After Nursing Home Admission: Racial and Ethnic Disparities and Risk Factors | 2017 | United States | New nursing home residents followed to 1-year | 15,927 (443) | None stated | Cohort | Home-level characteristics: percentage of residents receiving Medicaid, deficiencies in care quality (resident behavior–facility, practices–dignity, quality of care, and resident assessment and total number). Community-level: proportion of the Census tract community that was: American Indians, Asians, or Pacific Islanders, Black non-Hispanics, Hispanics, White non-Hispanics; percentage of Census tract below poverty level, percentage of Census tract residing in an urban area | Social engagement | The only significant predictor of low social engagement was at the community level: residing in a nursing home located in a community with a higher proportion of its population living an urban area (p=0.03). |
| DeBoer | Green Care Farms as Innovative Nursing Homes, Promoting Activities and Social Interaction for People with Dementia | 2017 | The Netherlands | Nursing home residents | 115 (18) | Inclusion: formal diagnosis of dementia according to medical record | Cross-sectional | Green care farms (vs traditional nursing homes and regular small-scale living facilities) | Social interaction | Compared to traditional nursing homes, residents of green care farms scored higher on social relations (p<0.05). No differences with regular small-scale living facilities were found. |
| DeBoer | Quality of care and quality of life of people with dementia living at green care farms: a cross-sectional study | 2017 | The Netherlands | Nursing home residents | 15 (18 nursing home wards/units | Inclusion: formal diagnosis of dementia according to medical record | Cohort | Green care farms (vs traditional nursing homes and regular small-scale living facilities) | Social engagement, social isolation and social relations | Residents of green care farms had significantly more social interaction (p=0.006) than residents of traditional nursing homes; the difference with regular small-scale homes was not statistically significant (p=0.429) |
| Hermer | Does Comprehensive Culture Change Adoption via the Household Model Enhance Nursing Home Residents’ Psychosocial Well-being? | 2017 | United States | Nursing home residents | 68 (3) | Exclusion: advanced dementia | Cross-sectional | Household model nursing home (vs. traditional nursing home setting) | Social interaction | Household model residents and staff spent more time in personal care sessions and other task-oriented interactions than did residents and staff at the control facilities. Some evidence of more time spent in non–task-oriented social interactions as well, but only a marginal difference across the three nursing homes. |
| Lood | The relative impact of symptoms, resident characteristics and features of nursing homes on residents’ participation in social occupations: Cross-sectional findings from U-Age Swenis | 2017 | Sweden | Nursing home residents | 4451 (up to 172) | None stated | Cross-sectional | Type of room (single or shared room) and type of unit (dementia unit or not) | Social engagement | In adjusted analysis, type of room did not predict participation in social occupations but residing in a dementia specific care unit was positively associated with participation in social occupations (p<0.01). |
| Boersma | Testing the Implementation of the Veder Contact Method: A Theatre-Based Communication Method in Dementia Care | 2018 | The Netherlands | Residents with dementia (n=141) and their professional caregivers (n=136) | 141 (4) | Inclusion: cognitive problems due to dementia | Pre-/post- | Veder contact method, a person-centered method using theatrical, poetic and musical communication for application in 24-hr care that encourages social interaction (vs. usual care) | Social relations and social isolation | Implementation of VCM led to significant positive improvements in the residents’ social relations (p=0.002). The association with social isolation was not statistically significant. |
| Kok | Quality of life in small-scaled homelike nursing homes: An 8-month controlled trial | 2018 | The Netherlands | Nursing home residents | 145 (2) | All residents had dementia | Pre-/post- | Large-scale living environment vs small-scale home-like unit | Social relations and social isolation | No significant differences were found between both groups on other aspects of quality of life |
| Gerritsen | Relationship of care staff attitudes with social well-being and challenging behavior of nursing home residents with dementia: a cross sectional study | 2019 | The Netherlands | Long-term care facility residents | 239 (15) | Residents had dementia | Cross-sectional | Nursing staff attitudes towards residents with dementia, including subscales for hope and person-centredness | Social well-being | When care staff had a more hopeful attitude towards residents with dementia, residents displayed higher social well-being (p <0.05, Model 3) but these effects were not found for ‘Person-centeredness’. |
| Wang | The Implementation of Restorative Care and Factors Associated with Resident Outcomes in Long-Term Care Facilities in Taiwan | 2019 | Taiwan | Long-term care facility residents | 210 (24) | None stated | Pre-/post- | Restorative care | Social support | There was significant improvement in social support (p<0.001); the main improvement in the social support domain was in emotional (p<0.001) and information (p=0.001) support. |
